# Thickness dependent oxidation in CrCl_3_: a scanning X-ray photoemission and Kelvin probe microscopies study

**DOI:** 10.3762/bjnano.16.58

**Published:** 2025-06-02

**Authors:** Shafaq Kazim, Rahul Parmar, Maryam Azizinia, Matteo Amati, Muhammad Rauf, Andrea Di Cicco, Seyed Javid Rezvani, Dario Mastrippolito, Luca Ottaviano, Tomasz Klimczuk, Luca Gregoratti, Roberto Gunnella

**Affiliations:** 1 Physics Division, School of Science and Technology, University of Camerino, 62032 Camerino (MC), Italy; 2 Elettra-Sincrotrone Trieste, Strada Statale 14, AREA Science Park, 34149 Trieste, Italy; 3 Dipartimento di Scienze Fisiche e Chimiche (DSFC) Università degli Studi dell’Aquila, Via Vetoio 10 67100 L’Aquila, Italy; 4 CNR-SPIN UoS L’Aquila. Via Vetoio 10 67100 L’Aquila, Italy; 5 Gdansk University of Technology, Faculty of Applied Physics and Mathematics, 80-233 Gdansk, Poland

**Keywords:** chemical mapping, CrX_3_, Kelvin probe force microscopy, mechanical exfoliation, scanning photoelectron microscopy (SPEM), two-dimensional material, work function

## Abstract

The modifications in the electronic properties induced by the thickness and size of an individual flake of transition-metal halides on different substrates (silicon oxide or In-doped tin oxide) are of particular technological interest, even more in the case of chromium trihalides (CrX_3_, X = Cl, Br, and I), whose longer lifetime under ambient conditions is particularly intriguing. By using synchrotron-based scanning photoelectron microscopy with a resolution of 0.1 μm and Kelvin probe force microscopy, we evaluated the surface modification reaction and the surface potential. Our results established the correlations of the two latter properties with the thickness of flakes, observing a natural tendency to preserve their characteristic when the flakes have significantly less thickness. This is in contrast to thicker flakes, which show alteration patterns similar to those observed in bulk-cleaved samples (Kazim, S.; Mastrippolito, D.; Moras, P.; Jugovac, M.; Klimczuk, T.; Ali, M.; Ottaviano, L.; Gunnella, R. *Phys. Chem. Chem. Phys.***2023**, *25*, 3806–3814. https://doi.org/10.1039%2FD2CP04586A%29. This preliminary study investigates interfaces made by dry transfer of CrCl_3_ flakes in an atmospheric environment. Cl vacancies and the formation of O/CrCl_3_ are induced, serving as dissociation centers that facilitate the migration of Cl vacancies between the top and bottom surfaces. By manipulating 2D atomic layers via surface oxidation or the introduction of surface vacancies, a novel and versatile approach is unveiled for the development of low-dimensional multifunctional nanodevices.

## Introduction

The family of chromium-based trihalides has garnered significant interest in recent years, particularly after the remarkable discovery of long-lasting magnetism in a single layer of CrI_3_ [1]. In our previous reports, we dealt with the environmental stability of CrCl_3_ [2], which can be easily exfoliated and exhibits a slower degradation rate compared to CrI_3_ or CrBr_3_[3]. To fully exploit the potential of any material, a detailed understanding of its electronic and structural changes arising from intrinsic and extrinsic defects is crucial [4]. Despite this importance, limited experimental research has been conducted on the electronic structure of CrX_3_ [5–6]. According to previously published photoelectron spectroscopy results, CrX_3_ belongs to metal compounds in which the 3d states are very close to the Fermi level, significantly above the 3p/4p/5p states of the halides. This has been supported by self-consistent band structure calculations by Antoci and Mihich [7], which introduced spin degeneracy, demonstrating that CrCl_3_ and CrBr_3_ behave as metallic system because of the prominent role of the 3d states near Fermi level. In our previous publications [8–10], we have found the formation of a stable and partially ordered Cr–O–Cl surface on vacuum- or air-cleaved CrCl_3_ samples. Building upon these findings, we now extend our chemical and structural investigations using electronically and optically characterized, mechanically cleaved CrCl_3_ samples [2,11,8,10], focusing on thin layered flakes and the role of the layer thicknesses obtained by spectro- and scanning microscopy with a lateral resolution of a few tens of nanometers. The interaction with the supporting substrate is a crucial factor [3,12] regarding the properties of the flakes, which can be significantly different from those of the bulk [13,1]. A central question remains whether modified structures and compositions arise from stress during cleaving, affecting surface terminations [10], or from the exfoliation process itself, which differs from cleaving. Regarding the bulk, we showed that oxygen adsorption on cleaved surfaces facilitates the formation of a stable structure with charge transfer signatures, as identified by high-resolution photoemission spectroscopy [8]. It remains unclear whether similar effects occur in exfoliated thin flakes. Like in other materials, the content of defects such as adatoms, the length of grain boundaries, vacancies, and substitution impurities influence the electrical, magnetic, and electronic properties of the final device [14–15,4]. To name one, the formation of chalcogenide vacancies is often related to the enhanced dissociation of molecular oxygen [16] at the metal species. These defects do not only change the electronic behavior of the sample by modifying the band structure [17]; they are also responsible for Curie temperature deviations, work function modifications [18], and induced long-range magnetic orders (i.e., magnetic band effect) [19–21]. A well-known and suitable technique to investigate the electronic structure of surfaces is X-ray photoemission spectro-microscopy [22,6]; in order to obtain the necessary spatial resolution, the beam size must be reduced to tens of nanometers. The Electron Spectroscopy for Chemical Analysis (ESCA) Microscopy beamline [23] enables this by means of a zone plate arrangement that can reduce the beam size to 130 nm in diameter, and its grazing collection angle can provide a highly surface-sensitive probing depth of approximately 1 nm [23–24]. Such a short mean free path condition could be suitable for increasing the sensitivity to a number of defects per unit volume forming at the surface, which can be recorded by the significant photoemission core level binding energy shift [25–26]. The significance of these studies lies in the exploration of novel materials with improved properties for 2D magnets by manipulating factors such as layer thickness, applied strain, and induced defect sites. Numerous theoretical studies predict that magnetic order in monolayers occurs at temperatures significantly higher than the bulk Curie temperature (i.e., 17 K). In their work, Liu et al. employed Monte Carlo methods to observe ferromagnetic behavior in monolayers below 66 K and proposed that hole doping could further enhance the Curie temperature [21]. Similarly, another Monte Carlo study found that the transition temperature for monolayer CrCl_3_ is 49 K, proposing that the Curie temperature could be further increased by applying uniaxial strain [17,27].

In the present study, we examined the surface modifications that occur in thin layers of exfoliated CrCl_3_ (approximately 1 to 20 ML) by using scanning photoelectron microscopy (SPEM). We collected the chemical maps and spectra of the Cl and Cr core levels at room temperature (RT). By monitoring the core levels and valence band spectra at various spatial resolutions (≥0.13 μm), we obtained quantitative maps of the chemical composition to correlate these maps with the thicknesses measured by AFM. Additionally, we investigated the correlation between the microscopic results and the surface potential of CrCl_3_ flakes at the nanoscale level using Kelvin probe force microscopy (KPFM) [28]. KPFM is mainly employed to measure the local contact potential difference between the conductive AFM tip and the sample, allowing for high-resolution mapping of the work function and surface atomic states [29]. This technique establishes a correlation between the valence band photoemission data and the morphological information, offering insights into the position of the conduction band [30].

## Results and Discussion

### Optical contrast and AFM

**Figure 1:**
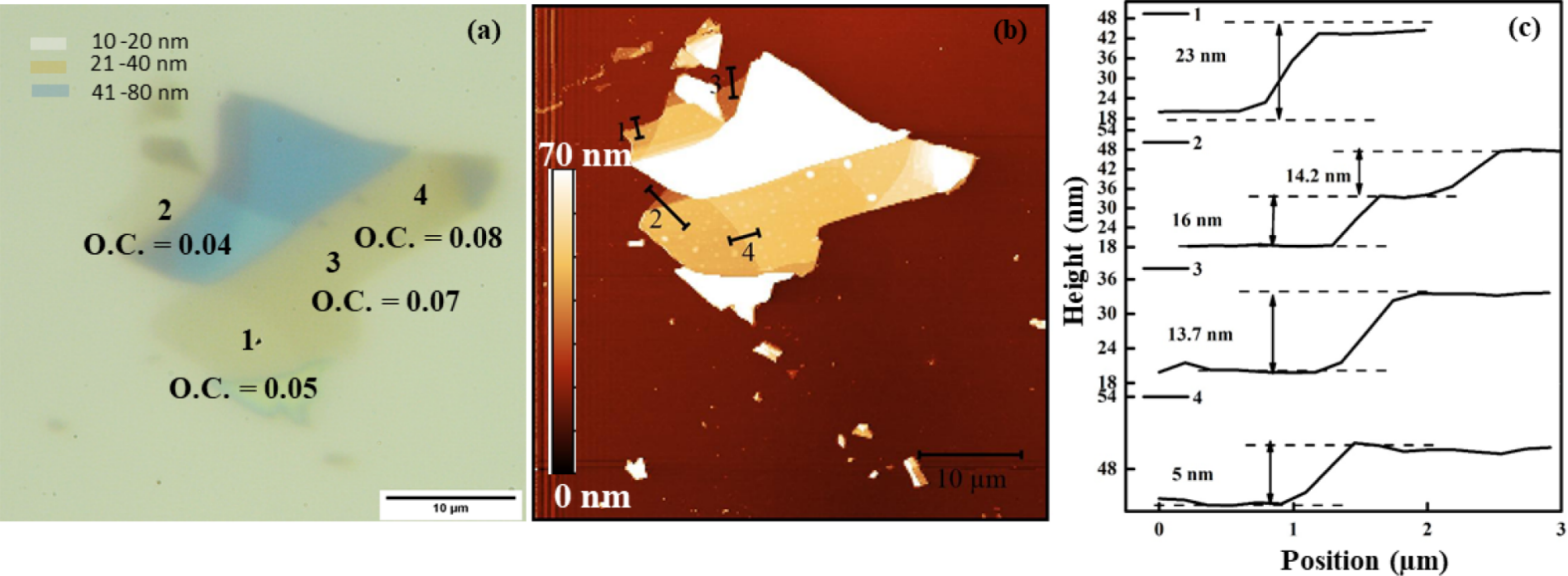
(a) Optical contrast and (b) AFM images of mechanically exfoliated CrCl_3_ flakes on the native Si (1 nm SiO_2_) substrate. (c) AFM thickness profile scans along the various flakes as denoted in Figure 1b.

**Figure 2:**
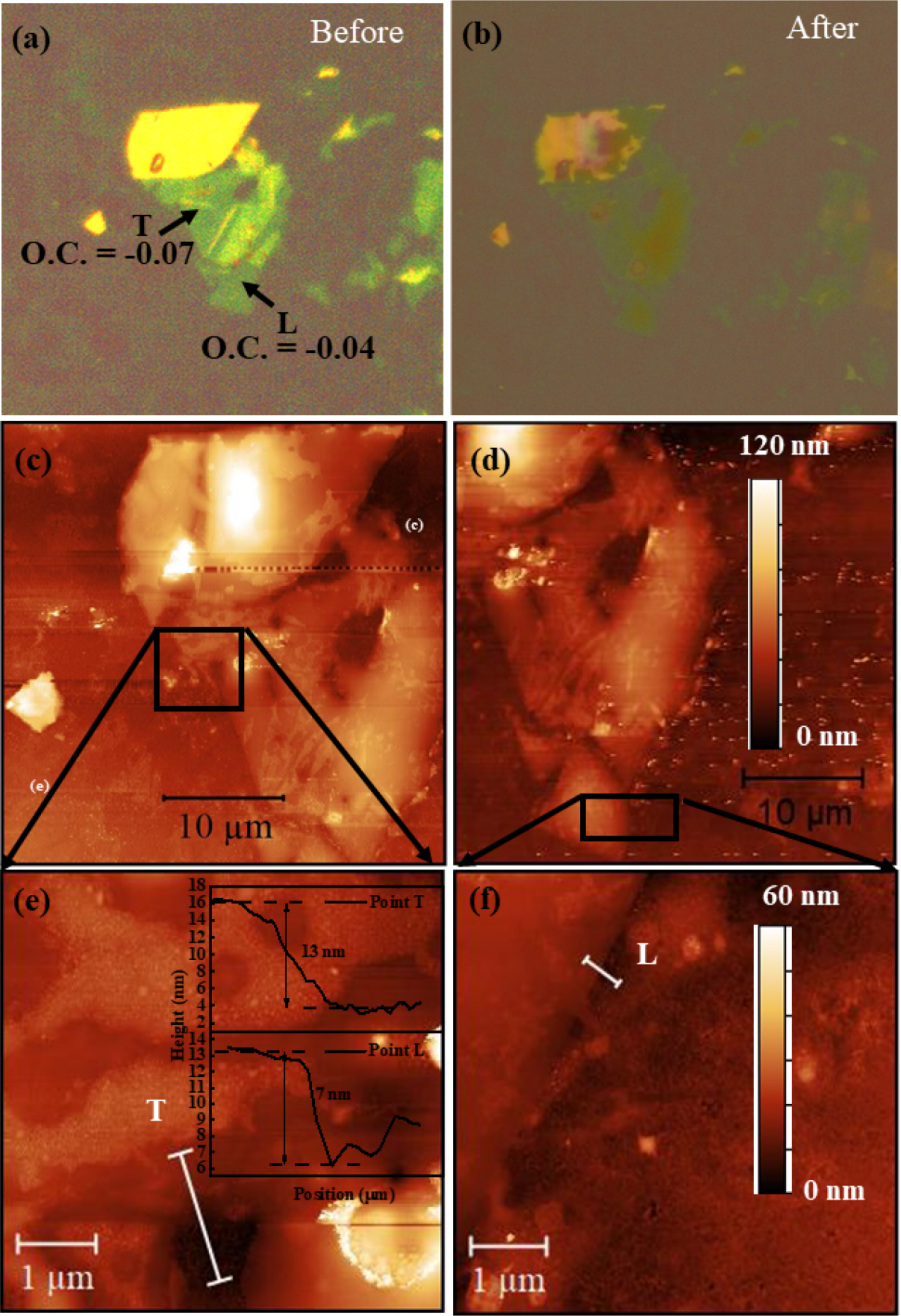
Optical contrast on ITO substrates (a) before and (b) after the SPEM measurements. (c, d) AFM topography images of CrCl_3_ on the ITO substrate for the points T and L in (a, b), respectively, together with zoomed details in (e) and (f). The inset displays the thickness profiles of T and L, respectively.

It is well known that 270 nm SiO_2_/Si substrates provide the highest optical contrast (O.C.) value for a single layer or few layers of CrCl_3_ [2,11]. Because of the insulating behavior of 270 nm SiO_2_ and to avoid surface charging, we utilized Si(001) wafers with a 1 nm layer of SiO_2_ and ITO films (190 nm) on glass substrates for SPEM investigations. In contrast, 285 nm SiO_2_/Si and ITO (190 nm) on glass substrates were used for surface potential studies. On native Si substrates, the optical contrast was insufficient to visualize thin flakes. We were unable to observe flakes with thicknesses smaller than 10 nm using the optical microscope. Therefore, we opted for an alternative substrate, indium tin oxide (ITO), to conduct the SPEM measurements on thinner layers. Figure 1 gives a direct comparison of AFM images and O.C. on the 1 nm SiO_2_/Si substrate. Optical contrast, AFM image, and a complete series of profiles showing layer thicknesses are given. To overcome the visibility barrier, systematic optical and AFM studies were performed for CrCl_3_ flakes on an ITO substrate and are reported for comparison in Figure 2a,b. Based on the colors of the flakes seen in the optical microscope images, an interval of thickness values for each flake could be determined. Since we have already mentioned that flakes thinner than 10 nm on 1 nm SiO_2_/Si substrates are hardly visible, we have defined the color range starting from 10 nm or higher.

In Figure 1, light olive indicates a thickness range of 10 to 20 nm, brown represents thicknesses from above 20 to 40 nm, and blue denotes thicknesses from 40 up to 80 nm. The tentative scale bar is presented in Figure 1a. Similar results, but with higher sensitivity, were obtained for the sample on the ITO substrate. The colors of the scale are given in Figure S1, Supporting Information File 1. For the latter sample, we distinguished lean (L) and thick (T) flakes by optical and AFM inspection (Figure 2). The Olympus B×60 system with objective lenses of magnifications of 5×, 10×, 20×, 50×, and 100× was used to find a color scale to interpolate the AFM thickness measurement results.

In Figure 1a and Figure 2a, we report a series of CrCl_3_ flakes of different thicknesses based on optical determination, and in Figure 1b and Figure 2c,d, we present the related AFM images to evaluate the thickness. Profile 3 in Figure 1b shows the minimum observable flake thickness of CrCl_3_, which yields a very fade contrast in the optical image in Figure 1a. In contrast, in Figure 2a, clear microscopy images of a few layers of flakes are shown. Based on the optical contrast value, L and T denote lean and thick steps, respectively, and the thickness variation has been confirmed through AFM images in Figure 2c–f. The negative value of optical contrast shows that the surface of CrCl_3_ looks brighter than the substrate. We show the thickness variation with AFM micrographs in the specific zoomed regions in Figure 2e,f, where the heights of the T and L profiles were measured and presented in inset of the Figure 2e.

### Spatially resolved photoemission

**Figure 3:**
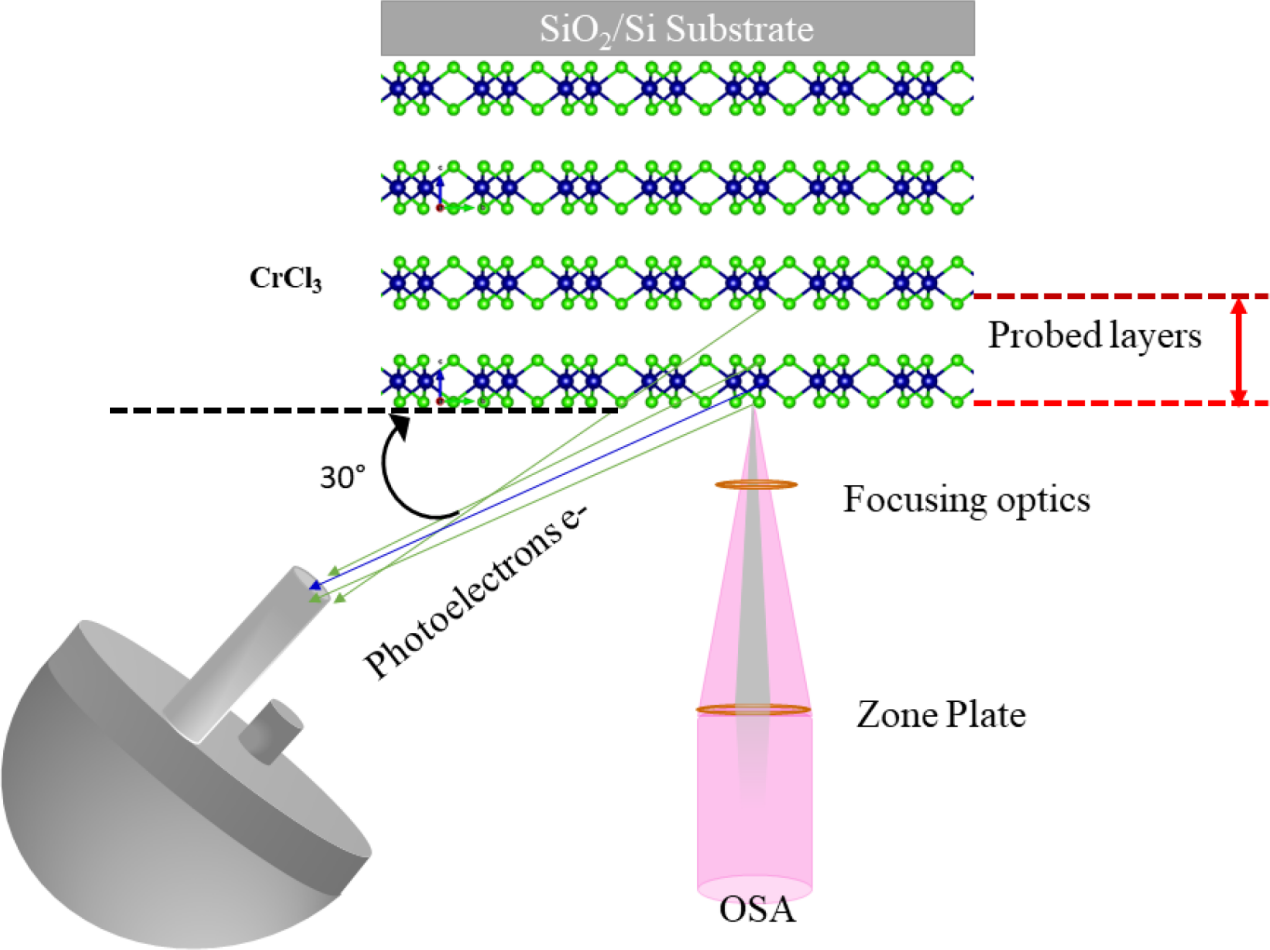
A schematic of experimental SPEM setup in the case of a few layer CrCl_3_ flake [27]. The lower part of the figure Figure 3 was redrawn from [31].

In our quest to understand exfoliated materials under varying photon flux conditions, we have extensively investigated the material while varying the incident photon energies [8]. As we aim for more ambitious goals, delving into variations of the material’s layers, ESCA microscopy emerges as a prime choice for making these groundbreaking experiments. We studied the layer-dependent surface modification of CrCl_3_ by SPEM, one of the optimal experimental techniques (Figure 3) for this kind of study. The use of zone plate and order selection aperture (OSA) provides an ideal spot size for the study of 2D materials, focusing on the character of the material in relation to its shape. Sample flakes in the range of 1–10 μm^2^, obtained by the exfoliation technique, can be analyzed by SPEM on such characteristic lengths by the precise control of the relative position between the beam and the sample. Thus, a determination of predominant phases without the influence of inhomogeneities or spurious effects (e.g., from the boundary of the flakes or different thicknesses) is possible. Furthermore, the very high background signal from the substrate can be minimized by the special design of the detection system [23].

**Figure 4:**
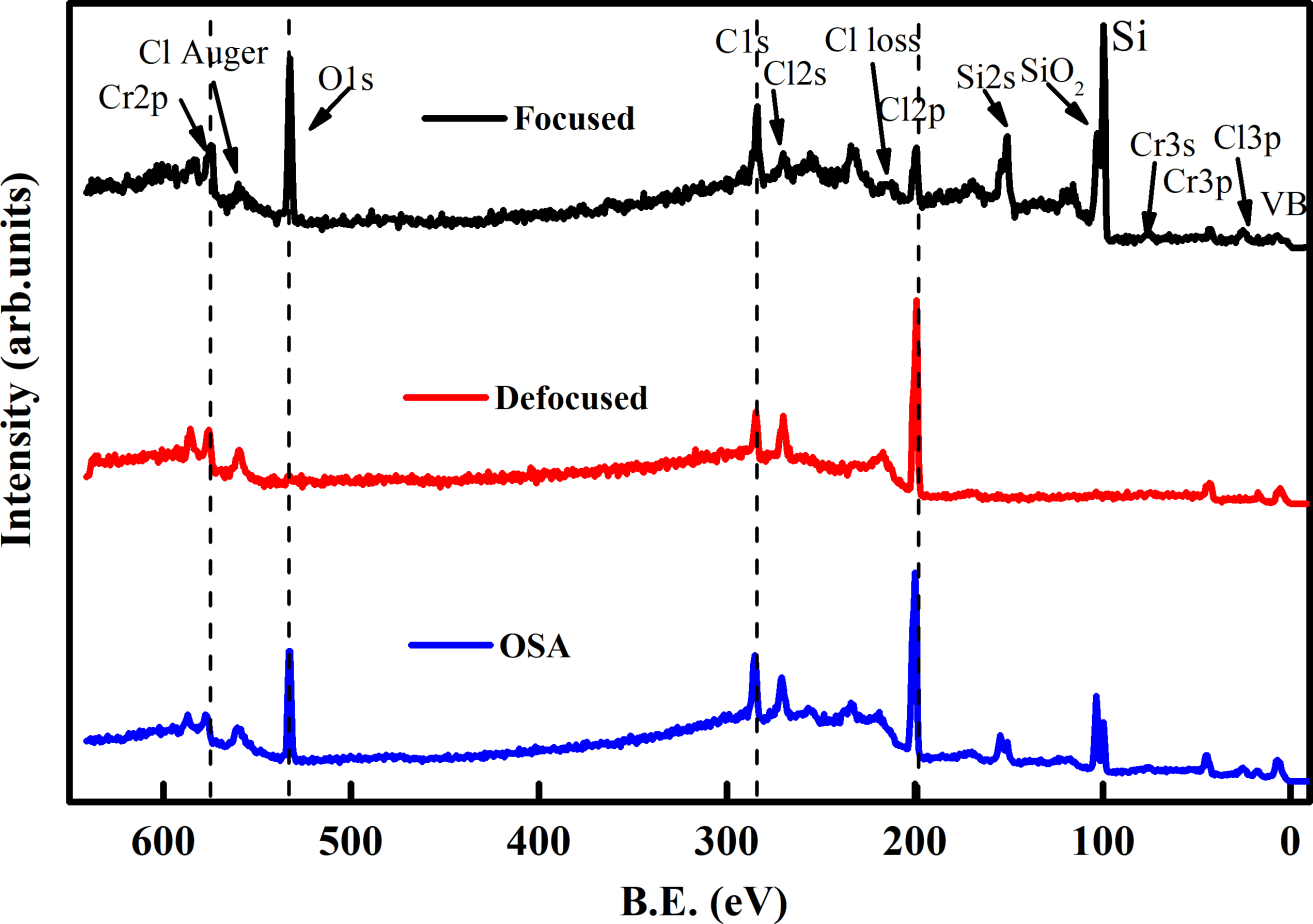
Survey spectra of exfoliated CrCl_3_ flakes on native Si oxide substrate with different beam modes at RT. From top to bottom: focused beam, defocused beam, and OSA mode of operation.

In SPEM, the spectra can be recorded in three modes, namely, (1) “focused” (beam size of ≈130 nm), (2) “unfocused” (beam size of ≈2 μm), and (3) the so-called OSA mode with a beam size of ≈75 μm. Figure 4 presents XPS survey spectra obtained with all three aforementioned modes on thin CrCl_3_ flakes at RT on a native Si oxide substrate. The survey spectra indicate beam damage resulting from the focused beam, as evidenced by the high intensity of the Si 2p core level photoemission, which was not observed in the other cases involving defocused beams. In contrast, the OSA measurement spectrum showed a Si component due to the wider beam diameter. This is further supported by the evaluation of the CrCl_3_ stoichiometry in the latter two cases.

Table 1 displays the area under the peaks for Cl 2p and Cr 2p. The stoichiometry was determined after applying the sensitivity correction factors. Under OSA beam conditions, the stoichiometry of CrCl_3_ exhibits the nominal value. However, there are significant deviations when measuring with a smaller beam size or for thin specimens exposed to high photon doses for several minutes. This behavior is corroborated by the signal from the Si 2p core level of the underlying substrate. When using the OSA mode, a contribution from the substrate is also anticipated because of the larger beam size.

**Table 1:** Stoichiometry determined from survey spectra using different photon beam sizes.

Beam mode	Cl 2p area	Cr 2p area	Cl/Cr ratio

focused beam	2190	4430	0.69
defocused beam	7500	5050	2.1
OSA beam	24900	11500	3.0

**Figure 5:**
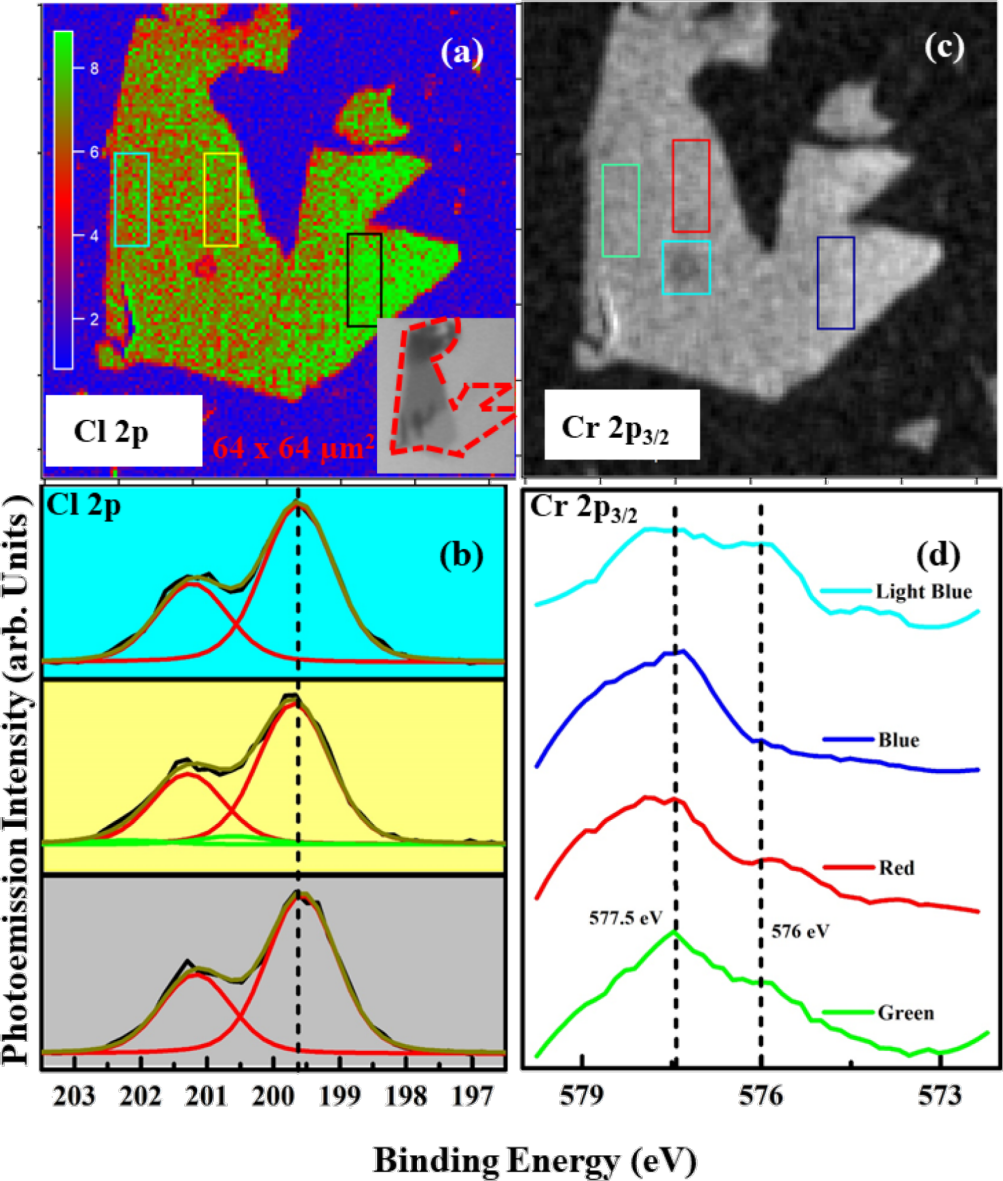
Cl 2p and Cr 2p_3_*_/_*_2_ core level SPEM maps with focused beam at RT on 1 nm SiO_2_/Si substrate. (a) Cl 2p map at 199.5 eV binding energy; (b) spectra with light blue, yellow, and dark gray backgrounds taken from the correspondingly colored rectangle areas in (a); (c) Cr 2p_3_*_/_*_2_ map at 577.5 eV binding energy; (d) binding energy spectra acquired from the correspondingly colored areas in (c).

During the mapping process, the sample was continuously scanned and data points were recorded within a few milliseconds. This method significantly reduced the incident photon dose compared to the survey spectra; however, it led to a lower statistical quality of the spectra. For further studies, we preferred to collect the core level spectra from maps using a defocused beam. In Figure 5, we have selected one particular flake consisting of two different main regions on the SiO_2_ (1 nm)/Si substrate. Figure 5a shows the Cl 2p map after background correction at a binding energy of about 200 ± 4 eV. We know from a previous work that at this energy the Cl 2p core level only shows the main component at 199.5 eV [8]. In contrast, in Figure 5c the Cr 2p_3_*_/_*_2_ spectrum shows also a second component at 576 eV in addition to the main component at 577.5 eV. Both maps display an increased intensity of Cl emission from the parts on the right side of the flake. On the left side, the density of Cl vacancies is supposedly higher (see inset of Figure 5a). In some recent works, the formation of a Cr–O–Cl surface phase [8,10] was characterized by the presence of a low-binding-energy component for Cl 2p at 198 eV (Figure 5b), albeit for a high degree of oxidation at high temperature in air, which we do not observe here, and a low-binding-energy component for Cr 2p_3_*_/_*_2_ at 576 eV (Figure 5d) from the presence of oxygen also in UHV. Such a deviation and component appearance is clearly visible in the thicker region of the sample (left region) for the Cr 2p core level. This component is also enhanced in the portion of the surface where beam effects were strong (light blue square in Figure 5c), but it is almost absent anywhere else.

**Figure 6:**
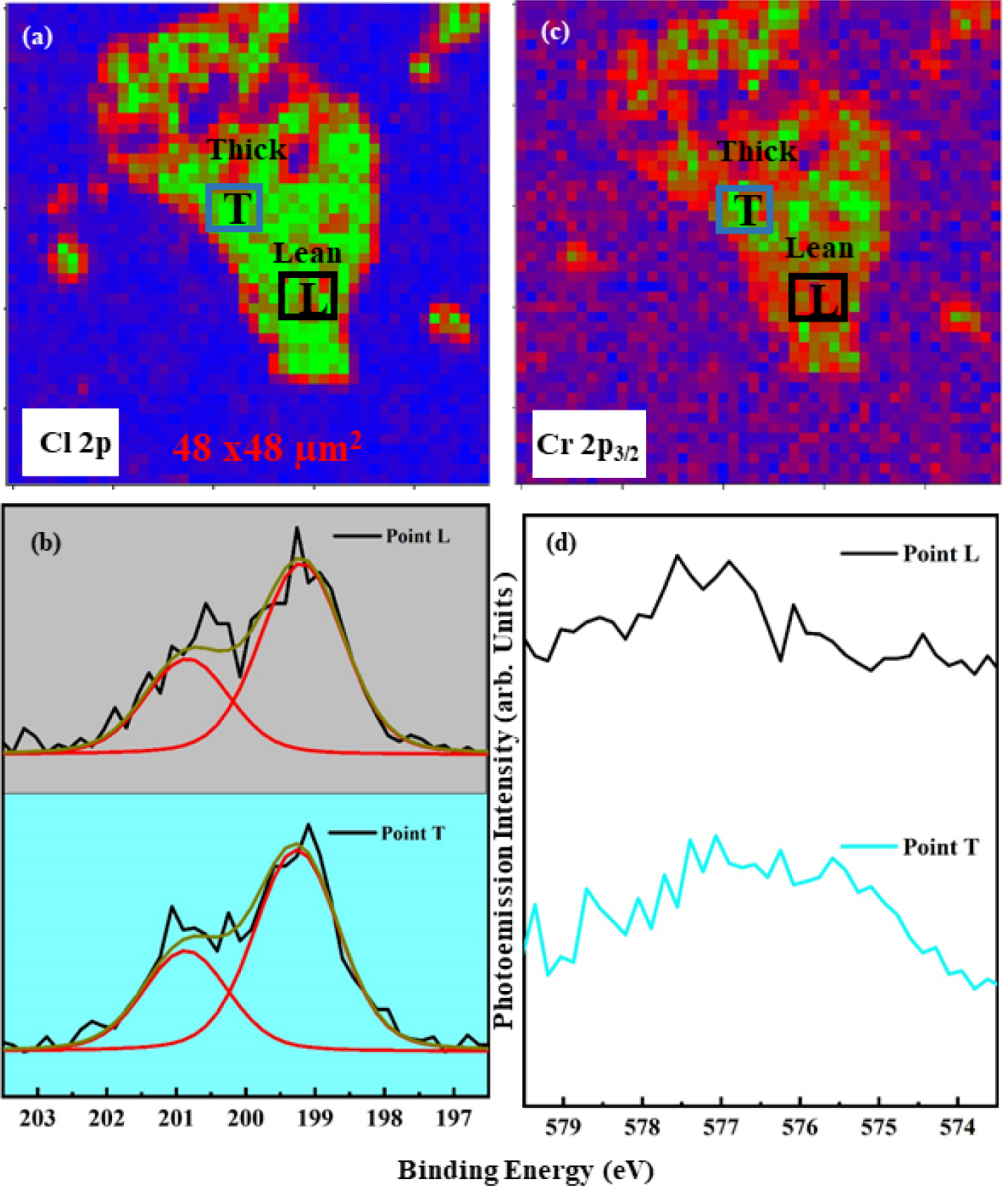
Cl 2p (a, b) (map and spectrum) and Cr 2p_3_*_/_*_2_ (c, d) (map and spectrum) core level maps at binding energies of about 200 and 576 eV, respectively.

**Figure 7:**
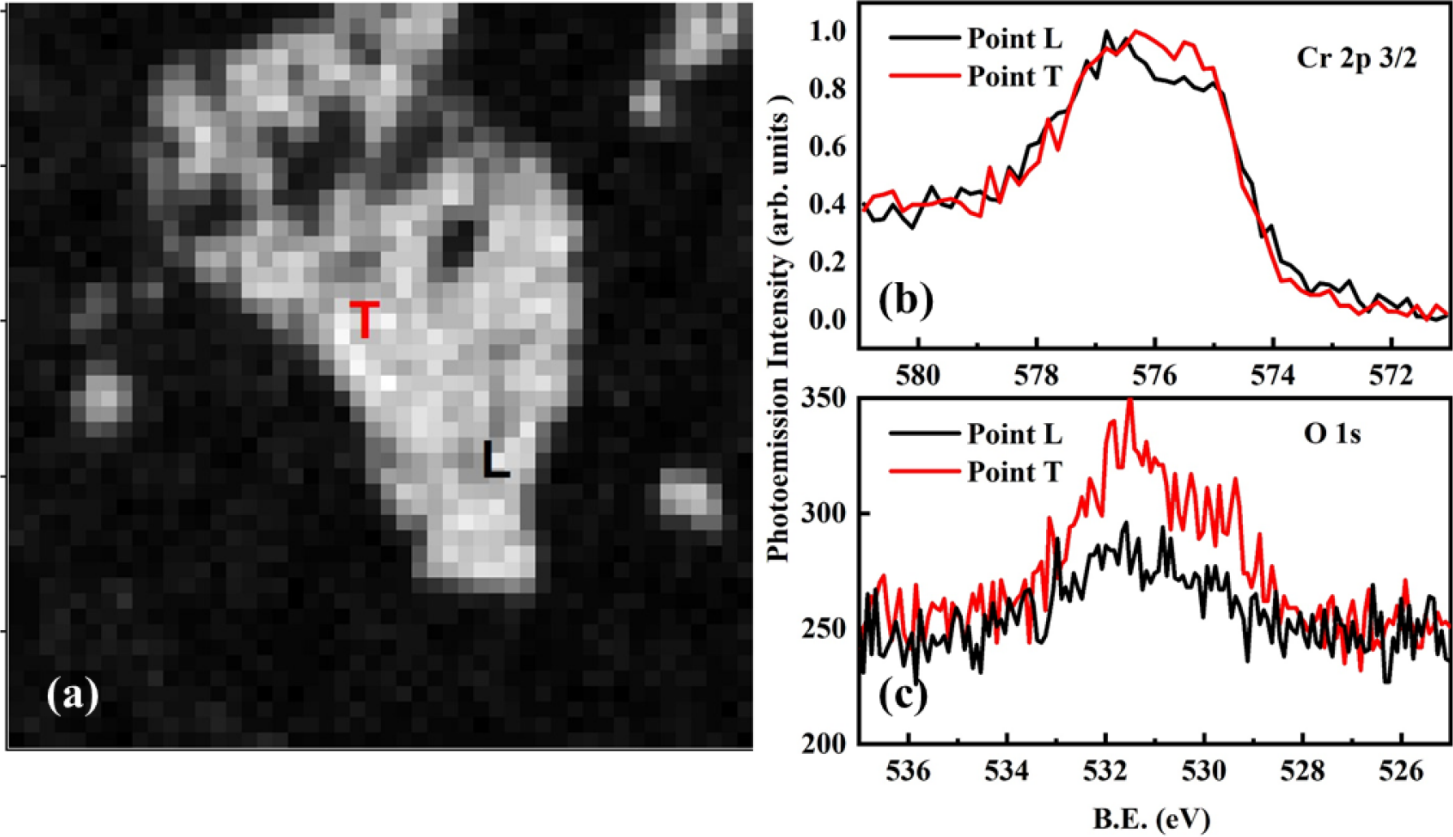
SPEM spectra from thick (T) and lean (L) areas on ITO. (a) SPEM image to verify the area of interest. (b) Cr 2p_3_*_/_*_2_ core level spectra. (c) O 1s core level spectra.

We also performed the core level analysis on the ITO substrate. Figure 6a shows the Cl 2p map around 199.5 eV binding energy. Leaner and thicker regions are designated with L and T, respectively. Figure 6b shows the Cl 2p spectra taken from the correspondingly colored regions after background correction. The upper spectrum was taken at point L, and the lower spectrum was taken at point T. The Cr 2p_3_*_/_*_2_ map is given in Figure 6c, and the corresponding Cr 2p_3_*_/_*_2_ core level data are shown in Figure 6d. The data were shifted vertically for better visualization. In the Cr 2p spectrum, one can clearly see the fingerprint of the low-binding-energy component for the thicker region (T), which is clearly absent in the leaner part (L). From our previous report, we know that the low-binding-energy component emerges after the formation of the O–CrCl_3_ phase [8]. To confirm our analysis, we have continued the investigation of the Cr 2p_3_*_/_*_2_ and O 1s core level spectra in Figure 7 at different thicknesses on the ITO substrate as reported in Figure 2 with defocused beam and observed the appearance of the peak at the lower binding energy of Cr 2p for the thick region, while the O 1s spectra appear to be enhanced for the same region.

These results can complement the study on cleaved samples [8], where the formation of a stable phase through oxygen in interstitial positions of the surface, which provided a redox source for Cr atoms, was shown by the formation of a low-binding-energy component at the Cr 2p core level. Here, this process occurs only in the presence of Cl vacancies and in the case of the thicker flakes. This lets us conclude that one of the reason of the resilience of the CrCl_3_ flakes is the fact that the formation of the Cr–O–Cl phase requires a sufficient volume of material, that is, that oxygen-driven phase formation is hindered if there are no specific diffusion processes in the sample. All three SPEM figures (Figure 5, Figure 6, and Figure 7) confirm that the lower-binding-energy component at 576 eV appeared because of the presence of Cl vacancies and the subsequent formation of reactive sites for the dissociation [16] of molecular oxygen to induce a stable phase of Cr–O–Cl [8]. We realized that the low-binding-energy component in Cr 2p_3_*_/_*_2_ is arising because of charge transfer effects. This turns the system from a surface Mott–Hubbard insulator to a charge-transfer [8] one in spite of Cr being an early transition metal [32].

What can be presumed from the present study is that Cl–O exchange following Cl vacancy formation is limited in very thin layers because of the limited diffusion processes and the lower number of defects present per unit volume that can be exploited in the process. These numbers are increased under beam irradiation, when damages occurs as shown by the light blue area in Figure 5. These results are consistent with previous studies on bulk samples cleaved in vacuum, where such a formation was very much hindered compared to thick flakes prepared in air [8].

It is nevertheless important to stress that here the modification is mostly driven by Cl vacancies alone. A strong evidence from this study and previous studies [8] is that only in case of bulk samples cleaved in air we observed the huge Cl vacancy signature in Cl 2p core levels (i.e., a low-energy component at 198 eV). Our conclusion is that the thin CrCl_3_ layers are more difficult to be modified because of the high energy of defect formation and the rapid quenching of them by mobile free atoms; this is confirmed by the high energy of Cl vacancies found by total energy calculations [9].

### Kelvin probe force microscopy (KPFM) measurements

**Figure 8:**
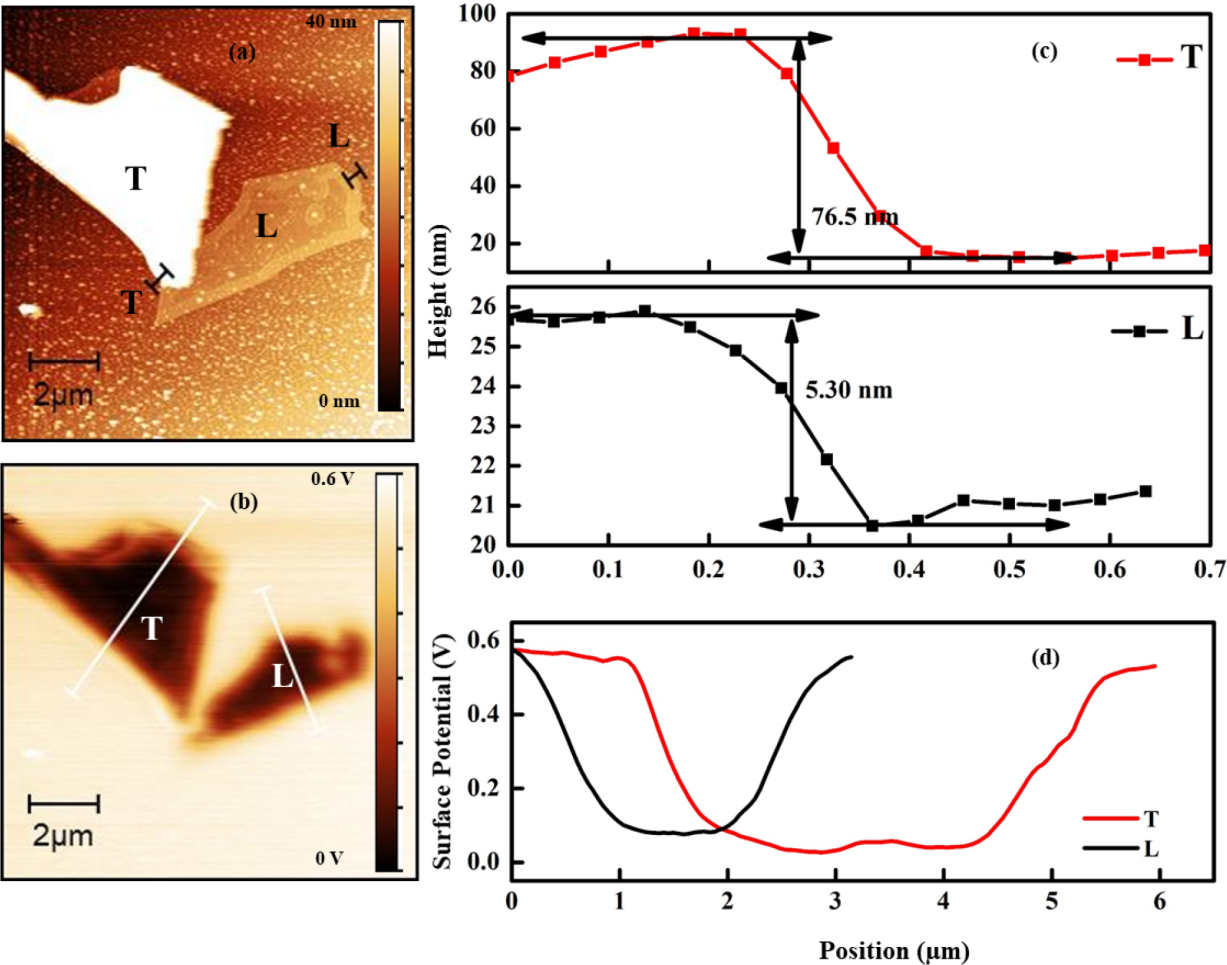
CrCl_3_ on SiO_2_/Si substrate. (a) Topography from non-contact mode AFM of two flakes with different thicknesses; (b) Kelvin surface potential of the samples on SiO_2_; (c) z-profiles of the two flakes; (d) Kelvin surface potential scans along the profiles of (a). A Pt-coated tip was used.

One look at the surface potential of the samples could complement our analysis. Figure 8a,c shows topographic maps and the corresponding thickness profiles of CrCl_3_ flakes on the SiO_2_ substrate. The average thicknesses of L and T CrCl_3_ flakes on the SiO_2_ substrate are about 5.3 and 76.5 nm, respectively. Figure 8b,d shows the Kelvin potential maps and the corresponding potential profiles. An obvious variation in the Kelvin potential of the flakes (*V*_KP_), that is, the difference between the surface potentials of tip and sample, could be observed, which is associated with the work function difference. In the *V*_KP_ line profiles, the flat region (at high potential) corresponds to the substrate, while the downward curved region (at low potential) is related to flakes. The *V*_KP_ values of L and T flakes are 0.10 and 0.04 V, respectively. Based on these values, the work functions of L and T flakes are about 5.40 and 5.46 eV, respectively, given the work function of the Pt tip of 5.50 eV. As a control, the work function of SiO_2_ was measured correctly as 5.00 eV.

**Figure 9:**
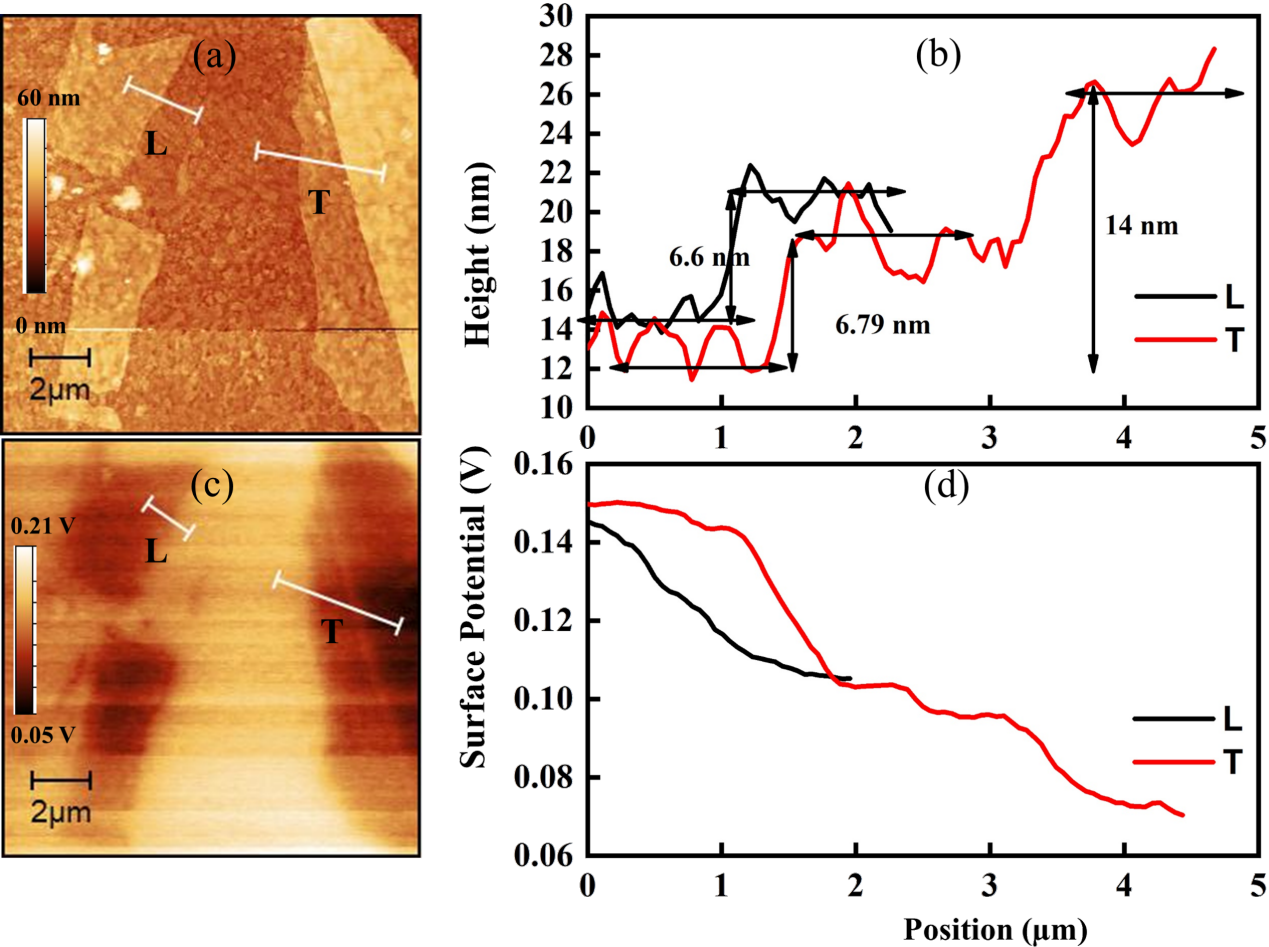
(a)Topography from non-contact mode AFM of flakes with two different thickness deposited on ITO; (b) Kelvin net surface potential of the samples in (a); (c) z-profiles of the two flakes; (d) Kelvin surface potential scans along the profiles of (a). A Pt-coated tip was used.

To complete the work begun with the spatially resolved photoemission measurements on the ITO substrate, for the obvious reasons related to the higher conductivity and a better contrast to individuate the flakes, we analyzed flakes in Figure 9, in which the thickness is plotted as a function of the position on the analyzed line. The work functions are 5.39 eV for flake L (thickness 6.6 nm) and 5.43 eV for flake T (thickness 14.0 nm), while 5.34 eV is the value for the ITO substrate. From the analysis of the surface potential on CrCl_3_ flakes presented in Figure 9, it can be seen that also in this case the areas with larger thickness have a higher surface potential, which is closer to the tip surface potential.

On both substrates, the KPFM results are similar, that is, the flake T has a higher work function than the flake L. This variation in the work functions of flakes is attributed to the chlorine vacancies in the thick region, which promote the chemisorption of oxygen and act as charge acceptor [33], but in a less efficient way with respect to Cl, as we observed by the low-binding-energy peak in the Cr 2p SPEM spectra. In contrast to physisorption, the chemisorption of oxygen has a significant impact on the electronic properties of a material. Neal et al. [34] reported the effect of chemisorption of oxygen as a kind of p-type doping, which shows consistency with our results. We expect the surface potential to show the same behavior depending on the chemical composition found by spatially resolved photoemission [35].

### Valence band results

**Figure 10:**
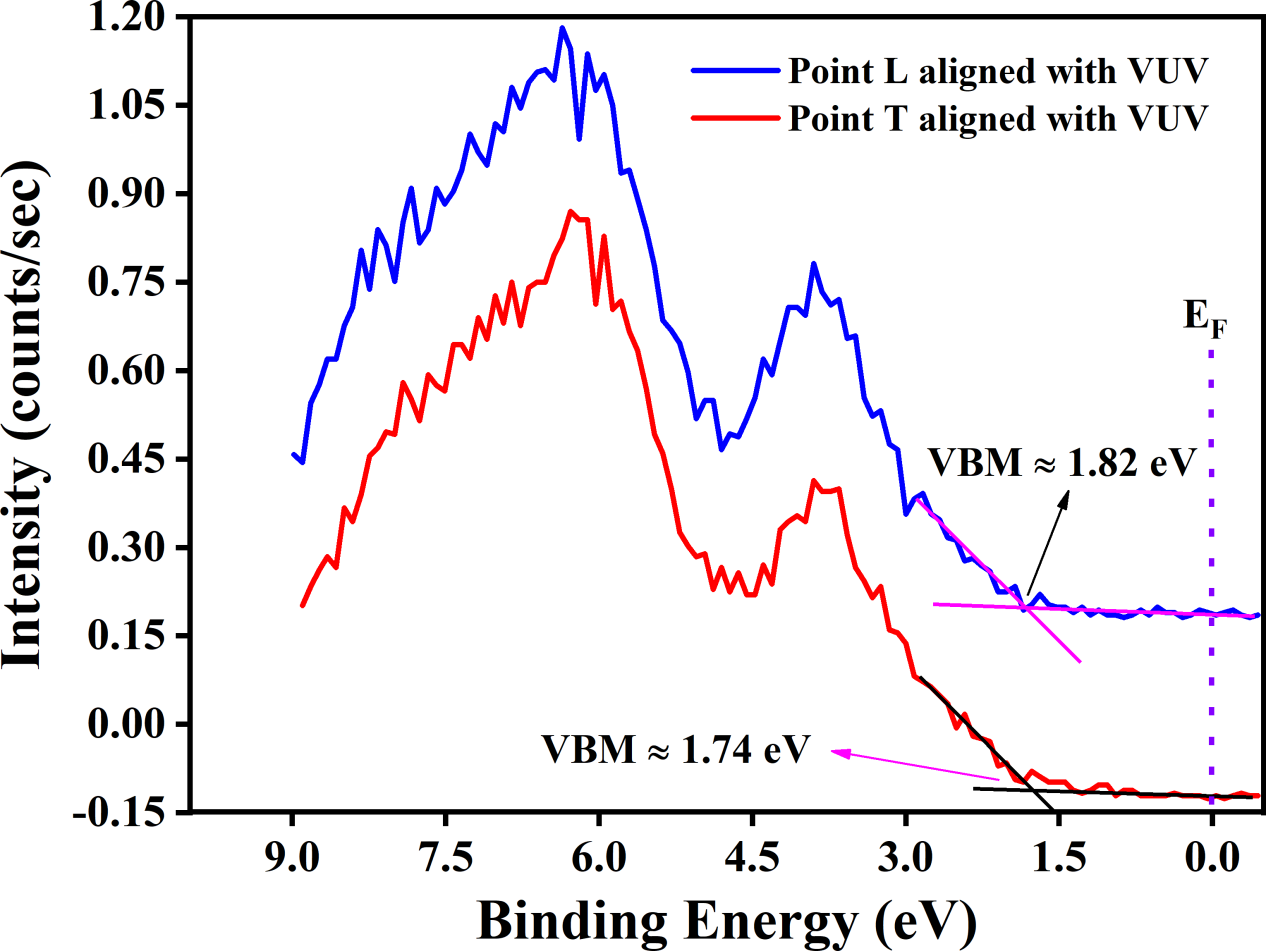
Valence band spectra acquired at point L (lean) and point T (thick). The spectra were aligned with respect to our previously published high-resolution photoemission spectra at 150 eV photon energy [8]. The alignment is shown in Supporting Information File 1, Figure S3.

The valence band spectra of CrCl_3_ flakes were recorded at the two different regions T and L. Figure 10 shows the valence band maxima (VBMs) for both regions. The VBM of point L is about 1.82 V, while for point T, it is about 1.74 V; the difference of 0.08 V is significantly above the limit of the experimental resolution. These VBM results reveal that point T is closer to the Fermi level than point L. This modulation of the Fermi level is primarily attributed to the existence of the Cr–O–Cl phase in the thick region. As reported in the KPFM results, the work function of point T is higher than that of point L. The difference between the work functions of these points in the KPFM results is about 0.06 V, which is consistent with the VBM results.

## Conclusion

**Figure 11:**
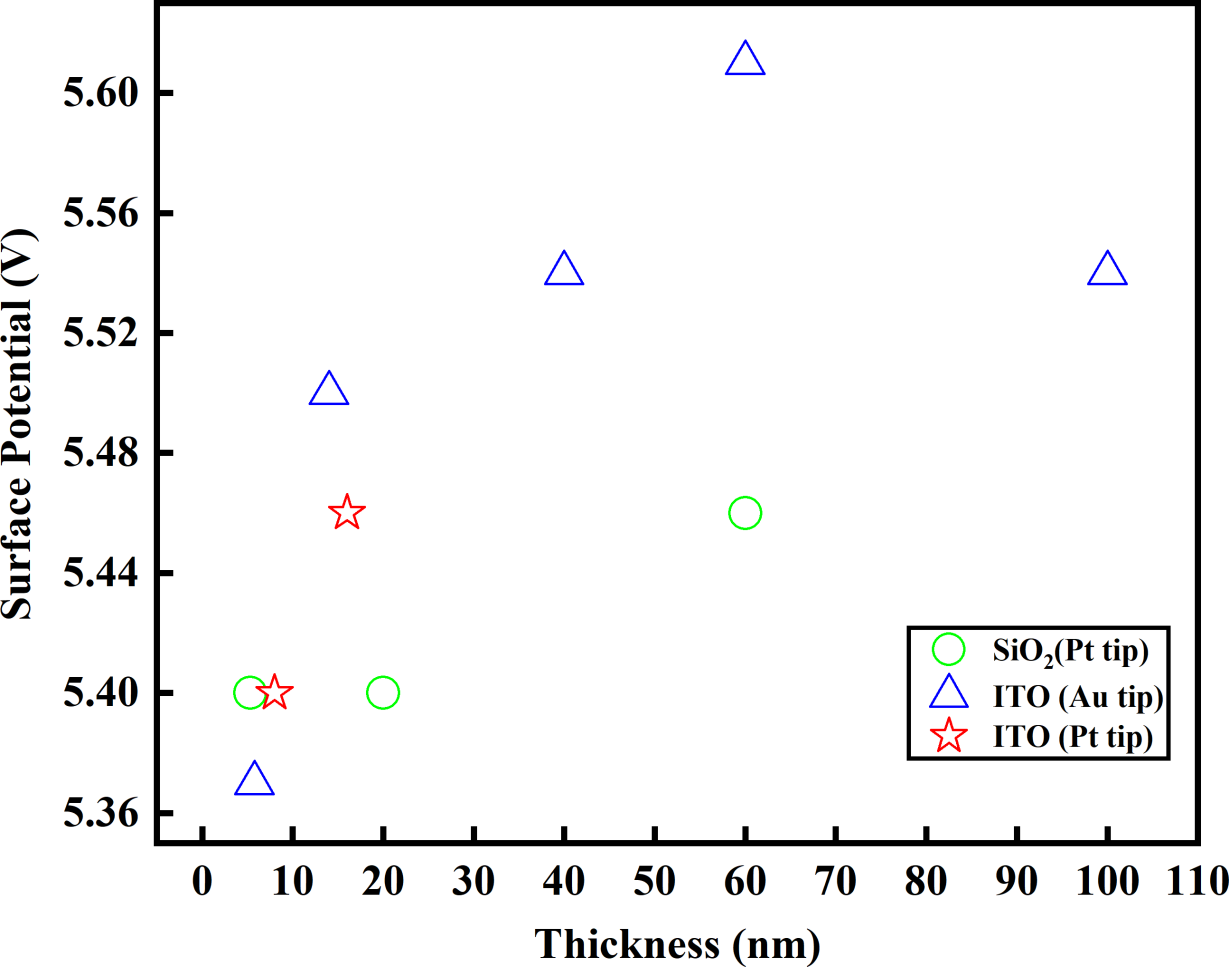
The surface potential values of CrCl_3_ flakes after several experiments as function of the flake thicknesses determined by AFM. Green: 285 nm SiO_2_ on Si substrate measured with Pt-coated tip (Kelvin potential 5.5 eV), red: ITO substrate (190 nm) on glass measured with a Pt-coated tip, and blue: ITO substrate (190 nm) on glass measured with a Au-coated tip (Kelvin potential 5.1 V).

Our work aimed at providing control of the surface evolution of thin specimens of CrCl_3_, contributing to establishing a method to engineer the material. In this specific case, it is difficult to find the reason of the composition modulation we have observed through the spatial resolved techniques. However, it could be the result of the combined effect of vacancies and a possibly induced dissociation of molecular oxygen. KPFM can be an interesting tool to describe the variation of chemical character of the 2D flakes with sizeable details. In Figure 11 we report the CrCl_3_ values of surface potential measured under different conditions on flakes of various thicknesses on SiO_2_ and ITO substrates. A steep increase of the surface potential represents the most evident variation on the CrCl_3_ surface, where a higher surface potential is related to a higher level of oxidation of the thicker flakes, probably related to an increased density of Cl vacancies acting as dissociation centers and the formation of a Cl-defective or O/CrCl_3_ surface structure [36,10]. Similarly to what was highlighted by the SPEM measurements, both measurements being surface-sensitive, the counter-intuitive finding of a lower degree of oxygen contamination is corroborating the conclusion that this behavior is a general trend for this material. This is an aspect of particular relevance because of the possible applications of the material to monolayer-thin devices.

## Experimental

The preparation of exfoliated CrCl_3_ flakes from the single crystal bulk material was reported in our previous papers [2,11]. Though preferentially a 270 nm silicon oxide substrate would help the determination of flakes thickness, we used also a more conductive substrate to measure photoemission under X-ray irradiation, that is, 1 nm thick native oxide Si substrates. Another convenient substrate for SPEM was 190 nm thick indium-doped tin oxide on glass, guaranteeing the necessary conductivity during the photoemission process. The 190 nm thickness was also important during the process of optical selection by showing significant contrast in an optical microscope (×50 magnification), helping a better localization of thin flakes. The SPEM measurements were performed at the ESCA Microscopy beamline 2.2L of Elettra Synchrotron Trieste facility, Italy. The incident photon energy of ≈740 eV was calibrated by means of Au f_7_*_/_*_2_ at 84.0 eV from a clean gold foil sample. To reduce the beam-induced effects on the samples, we recorded the high-energy-resolution spectra with an unfocused beam (≈2.0 μm diameter), while the high-resolution SPEM maps of 128 × 128 μm^2^ size using a piezoelectric driven stage were obtained with a focused beam (pixel size of 130 nm) by means of a Fresnel zone and a relatively broad energy resolution mode [23]. The SPEM maps were captured through a 48-channel delay line detector.

To analyze the photoelectron intensity of an individual atomic element on the captured SPEM maps, the image underwent background correction by eliminating the topographic features. We also applied the (3 × 3) filter to reduce the noise before extracting the photoemission spectra from the particular SPEM map. Figure 3 shows the schematic setup of the focusing optics and the hemispherical photoelectrons detector arrangement of the SPEM system.

Atomic force microscopy images were acquired with the NanoObserver (CSI) AFM system in resonant mode using an n-type Si cantilever coated with Pt at the resonance frequency of 68 kHz with an elastic constant of 1–5 N/m (AppNano) and doped diamond tips with 120 kHz and 8 N/m elastic constant (ADAMA). Kelvin probe force microscopy images were taken via double passage before and after applying an electric field by elevating the tip about 50–150 nm to measure the surface potential and avoid the influence of morphological features. The applied voltage was varied from 0.2 to 1 V without significant changes in the measured surface potential value. All micrographs were recorded at room temperature.

## Supporting Information

File 1 Technical details.

## Data Availability

Data generated and analyzed during this study is available from the corresponding author upon reasonable request.
