# Supplementary material for: Thickness dependent oxidation in CrCl3: a scanning X-ray photoemission and Kelvin probe microscopies study
Source: Beilstein J Nanotechnol. 2025 Jun 2;16:749–61. doi: 10.3762/bjnano.16.58 (PMC12152317; doi:10.3762/bjnano.16.58)
Supplement: File 1 — Technical details. [file Beilstein_J_Nanotechnol-16-749-s001.pdf]

BEILSTEIN JOURNAL OF NANOTECHNOLOGY

Supporting Information

for

Thickness dependent oxidation in $CrCl_3$: a scanning X-ray photoemission and Kelvin probe microscopies study

Shafaq Kazim, Rahul Parmar, Maryam Azizinia, Matteo Amati, Muhammad Rauf, Andrea Di Cicco, Seyed Javid Rezvani, Dario Mastrippolito, Luca Ottaviano, Tomasz Klimczuk, Luca Gregoratti and Roberto Gunnella

Beilstein J. Nanotechnol. **2025,** *16,* 749–761. doi:10.3762/bjnano.16.58

Technical details

License and Terms: This is a supporting information file under the terms of the Creative Commons Attribution License (https://creativecommons.org/licenses/by/4.0). Please note that the reuse, redistribution and reproduction in particular requires that the author(s) and source are credited and that individual graphics may be subject to special legal provisions.
The license is subject to the *Beilstein Journal of Nanotechnology* terms and conditions: (https://www.beilstein-journals.org/bjnano/terms)

Thickness approximation through optical images:

On ITO substrate, Fig.S1, the defined colour scale is as follows: green colour corresponds to 3 nm to 15 nm thick flakes, yellow colour corresponds to 15 nm to 40 nm thick flakes, while pink and violet colour represent the range of 40-80 nm and 80-120 nm thick flakes, respectively.

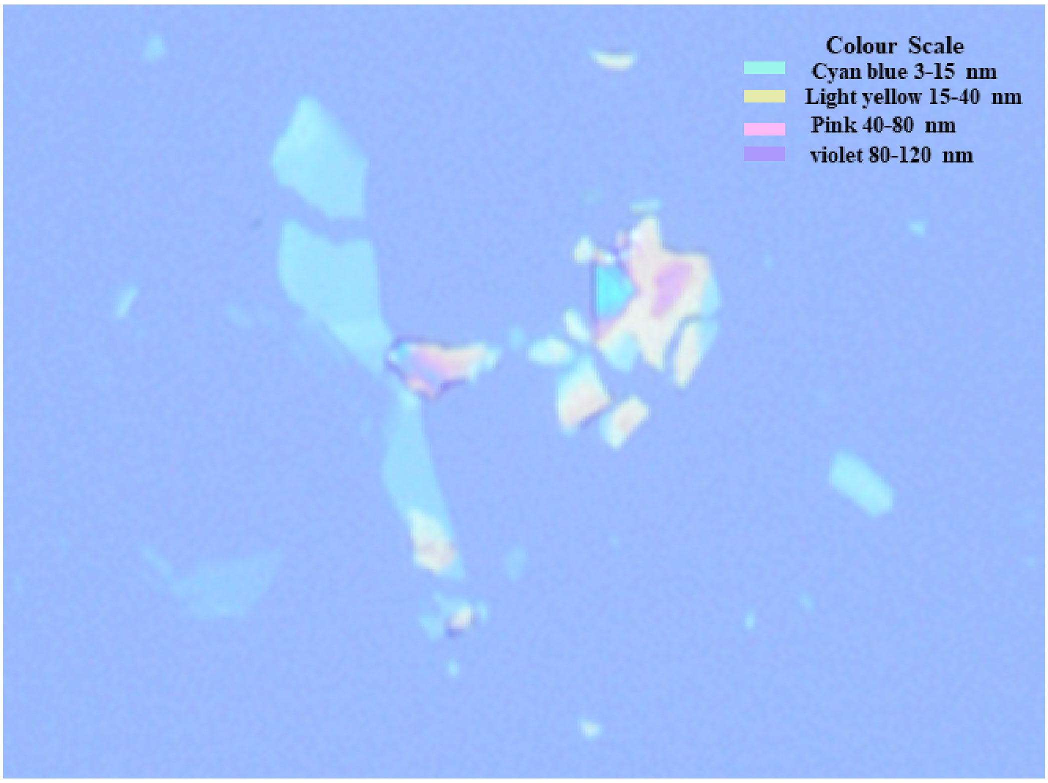

Figure S1: Optical microscope image of $CrCl_3$ flakes acquired with 20× magnification objective lens showing the multiple thick layers where each color is corresponding to a particular thickness value $CrCl_3$ flakes on ITO substrate.

Survey spectra on ITO substrate:

For samples grown on ITO substrates, survey spectra (beam size ≈2 µm) of point L and point T are reported in the Fig. S2 in Supplementary material, show a smaller contribution of oxygen at the surface. In the thinner region, the oxygen contribution is a little lower than in the thicker region to confirm that thin sample are more stable and less affected by rearrangement of the composition.

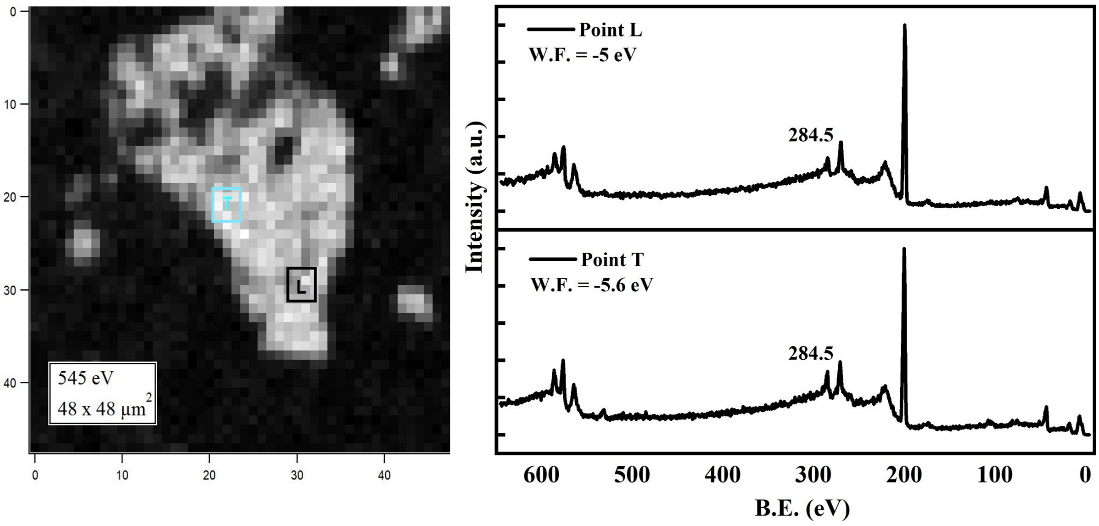

Figure S2: (a) Cl 2p map to represents the exact point positions for corresponding survey spectra presented in (b).(b) Survey spectra

Valence Band alignment:

To align the valence band spectra across varying thicknesses of $CrCl_3$ specimens, we opted to utilize high-resolution photoemission spectra from our prior investigation[1]. The red spectrum illustrates the valence band spectrum obtained with a photon energy of 150 eV incident on the $CrCl_3$ sample. Meanwhile, the black and green spectra represent the valence band spectra of lean (Point L) and thick (Point T) flakes, respectively.

Working Principle of KFM:

The surface topography and local work function of $CrCl_3$ were elaborated using the multimode AFM (Concept Scientific Instrumentation (CSI)) under the ambient conditions. These analyses were carried out using Pt coated n-Si tip (ϕ_{Pt} = 5.5 eV, frequency 43 - 81 kHz, spring constant 1 - 5 N/m) and boron doped diamond tip (Φ_{dia} = 5.1 eV, frequency 50 - 150 kHz, spring constant 8(4-16) N/m). The surface morphology of the flake was mapped using the resonant mode, while the KFM measurements were carried out using the double pass mode. Generally, the local electrostatic

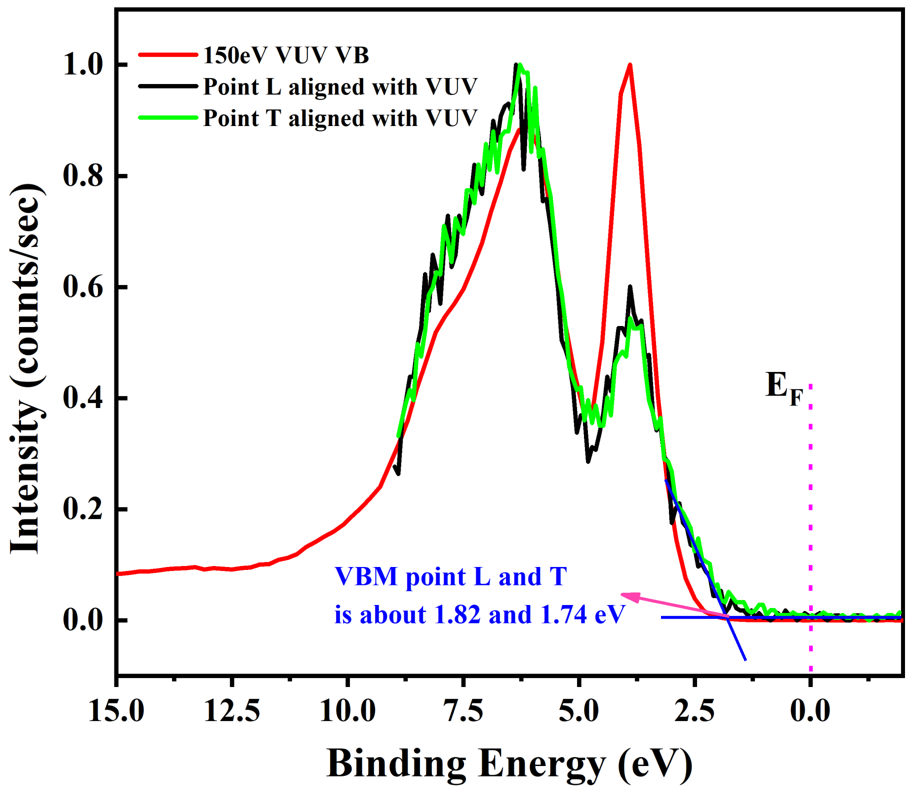

Figure S3: The valence band spectra : The red curve represents the reference spectrum obtained at a photon energy of 150 eV[1]. The black spectrum corresponds to the spectrum acquired from Point L, while the green spectrum corresponds to the spectrum obtained from Point T.

interaction of cantilever with a sample can be minimized in resonance mode (AM-AFM). In this mode, the tip scans twice the same line. During the first pass, the tip scan sample in x-y plane and record the surface topography without applied bias voltage. In the second pass, the cantilever raised to user-defined height to overcome the topographic effect due to van der Waals interaction at short range [2] following the first scan. The KFM micrograph is obtained by applying simultaneously AC $V_{AC}\, sin(\omega t)$ and DC V_{DC} biases [3] to measure the local electrostatic potential between the tip and sample.

When two metals (i.e., tip and sample) are brought into contact, electrons flow from low work function (high Fermi energy level) to high work function (low Fermi energy level) regions, which produce an opposite charge on both surfaces and consequently lead to contact potential difference (CPD) or Kelvin potential (V_{KP}) difference generation and alignment of Fermi levels, while the vacuum levels are no more aligned [3]. The magnitude of V_{KP} depends on the tip-sample work

function difference. To obtain the V_{KP}, both AC and DC biases are applied between the tip and sample. In this mode, the tip-sample configuration is considered as a parallel plate capacitor, the electrostatic force between the tip and sample can be expressed,

$$F_{es} = -\frac{1}{2}\frac{dC}{dz}V^2 \tag{S1}$$

$$F_{es} = -\frac{1}{2}\frac{dC}{dz}[(V_{DC} - V_{CPD}) + V_{ac}sin(\omega_{AC}t)]^2 \tag{S2}$$

Where C is the differential capacitance and changes due to the tip oscillations, V is the Kelvin potential (V_{KP}), and Z is the tip-sample distance. F_{es} can yield three components, wherein the F_ω which is the first harmonic component of F_{es} depends on the CPD,

$$F_\omega = -\frac{dC}{dz}(V_{DC} - V_{CPD})V_{ac}sin(\omega_{AC}t) \tag{S3}$$

By manipulating various force terms that can be cancelled out through adjustments in the DC voltage (resulting in V_{DC}), and utilizing the AC voltage as feedback to enhance sensitivity towards zero force, a distinction between Kelvin voltage (V_{KP}) and contact potential voltage (V_{CPD}) becomes evident. It is important to clarify that while V_{CPD} and V_{KP} are often used interchangeably, for the sake of clarity, we define V_{CPD} as the potential difference that emerges when materials with different work functions (such as the AFM tip and sample) come into contact. This represents the actual physical potential difference resulting from work-function disparities. On the other hand, V_{KP} denotes the applied compensation voltage, with V_{CPD} serving as its physical basis. The resulting vibration of the cantilever is detected using the same system of the AFM through a four-quadrant detector. A null circuit voltage is used to drive the DC potential of the tip to a value which allows to detect the surface potential when by a lock-in technique the double-frequency AC component is

filtered out. A map of this nulling DC potential versus the lateral position coordinate, therefore, produces an image of the work function of the surface. The measured potential and the work function of the sample are connected by the relation: $V_{Kelvin} = (\phi_{tip} - \phi_{sample})/e$ Where Φ_t is the work function of tip and ϕ_s is the work function of sample. The latter measurement is done after the tip is shifted by a definite amount along z to keep a constant distance between the sample and tip, to avoid side capacitance effect, and to favour better single-noise ratio[4]. In this case, the effective tip-sample distance is between 30 and 200 nm. In order to study the behavior of the work function of $CrCl_3$ flakes based on their thickness, we exfoliated various samples on ITO substrate and analyzed them with KFM. Fig.S4 shows an optical microscope image of the area where analyses are reported below. As you can see there are flakes of several colours shown in the colour scale previously reported for the Olympus BH2-UMA microscope, this means that in this area there are flakes with a thickness ranging from about 3 nm to more than 100 nm. Obviously, since the image was taken with a magnitude of 20x, it does not provide the detailed thickness for each area; for this purpose, data will be analysed with the KFM. Below are the topography and surface potential images of the most interesting areas for the purposes of our survey. In particular we want to identify the role of the surface potential not only where the samples show different thickness of the flake but also aim at looking to the behavior close to defects. For the calculation of the work function, it must be taken into consideration that all the data were taken using the Pt (5.5 eV) and the diamond tip of the KFM, with a Work Function of 5.1 eV [5].

The DC voltage value which has to be set to start the measurement was fixed at 0.3 V for all the measurements, as the best results were obtained with this value. The distance between the tip and the sample was instead varied for each flake to allow the surface potential to be high quality and not influenced by the topography for all the measurements but ranging from 30 to 200 nm.

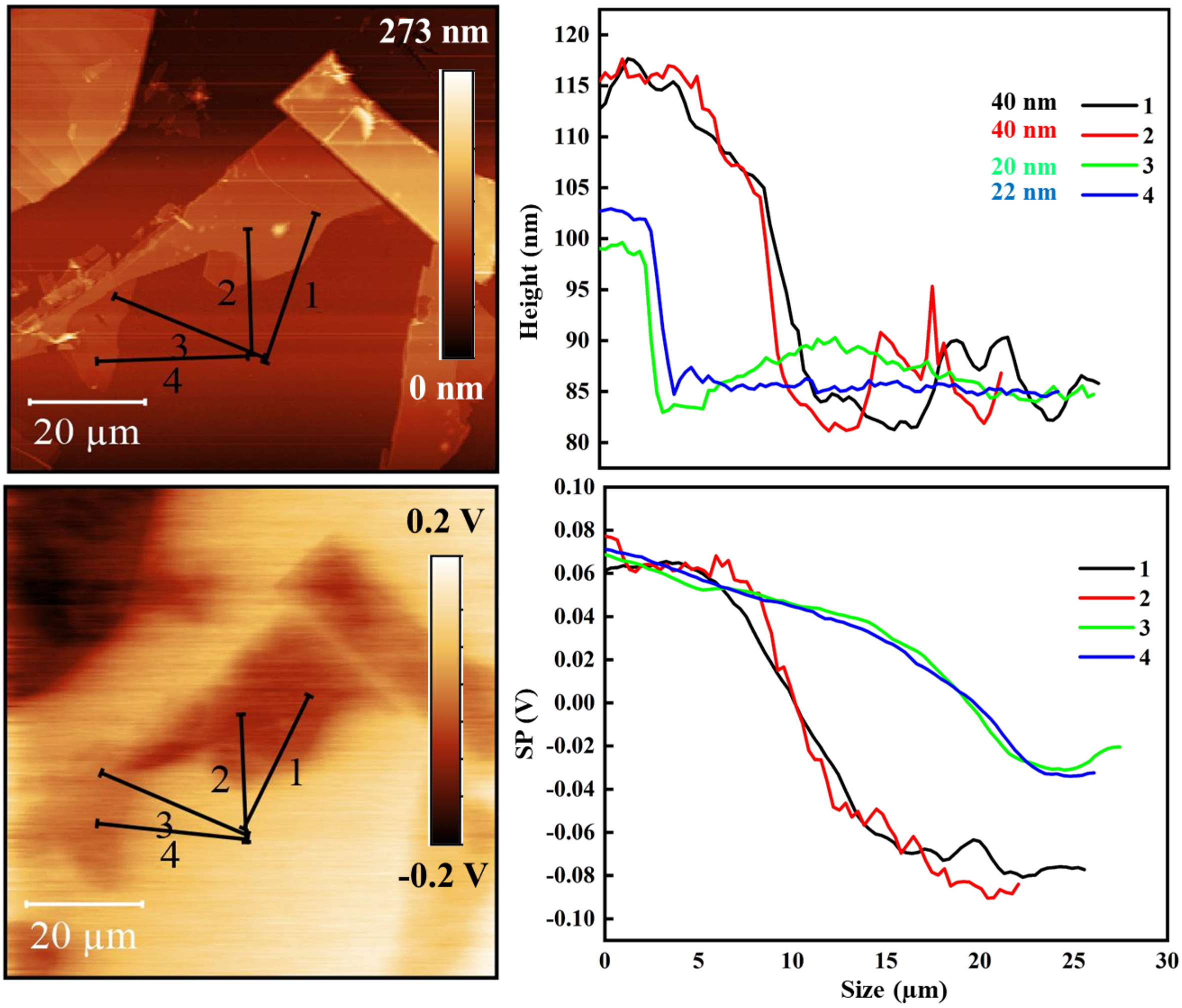

Figure S4: a) Topography by non-contact mode AFM on several thickness flakes deposited on ITO taken with a Au tip; b) Kelvin surface potential of the samples in a); c) z-profiles of the two flakes; d) Kelvin surface potential scans along the profiles.

References

1. Kazim, S.; Mastrippolito, D.; Moras, P.; Jugovac, M.; Klimczuk, T.; Ali, M.; Ottaviano, L.; Gunnella, R. *Physical Chemistry Chemical Physics* **2023**. doi:10.1039/D2CP04586A.

2. Xiaotian Zhu, G. H. t. B. B. J. K., Lijuan Xing; Palasantzas, G. *The Journal of Physical Chemistry C* **2021**, *125* (23), 12870–12879. doi:10.1021/acs.jpcc.1c02079.

3. Melitz, W.; Shen, J.; Kummel, A. C.; Lee, S. *Surface science reports* **2011**, *66* (1), 1–27. doi:10.1016/j.surfrep.2010.10.001.

4. Borowik, Ł.; Kusiaku, K.; Théron, D.; Mélin, T. *Applied Physics Letters* **2010**, *96* (10), year.

5. Ghomi, S. Chemical vapor deposition of ultra-thin molybdenum ditelluride films and their morphological and optical characterization. Ph. D. Thesis, Politecnico di Torino, 2021.
